# Dynamic evaluation of blood immune cells predictive of response to immune checkpoint inhibitors in NSCLC by multicolor spectrum flow cytometry

**DOI:** 10.3389/fimmu.2023.1206631

**Published:** 2023-08-10

**Authors:** Weijie Ma, Sixi Wei, Siqi Long, Eddie C. Tian, Bridget McLaughlin, Maria Jaimes, Dennis J. Montoya, Varun R. Viswanath, Jeremy Chien, Qianjun Zhang, Jonathan E. Van Dyke, Shuai Chen, Tianhong Li

**Affiliations:** ^1^ Division of Hematology/Oncology, Department of Internal Medicine, University of California Davis Comprehensive Cancer Center, University of California Davis School of Medicine, Sacramento, CA, United States; ^2^ Department of Pathology and Laboratory Medicine, Dartmouth-Hitchcock Medical Center, Geisel School of Medicine, Dartmouth, NH, United States; ^3^ University of California Davis, Flow cytometry Shared Resource, Davis, CA, United States; ^4^ Cytek Biosciences, Fremont, CA, United States; ^5^ Department of Biochemistry and Molecular Medicine, University of California Davis, Sacramento, CA, United States; ^6^ Beckman Coulter Life Sciences, San Jose, CA, United States; ^7^ Division of Biostatistics, Department of Public Health Sciences, University of California, Davis, Davis, CA, United States; ^8^ Medical Service, Hematology and Oncology, Veterans Affairs Northern California Health Care System, Mather, CA, United States

**Keywords:** immune biomarker, blood, predictive, multiplex, peripheral blood mononuclear cells, immune checkpoint inhibitors, spectrum flow cytometry, non-small cell lung cancer

## Abstract

**Introduction:**

Immune checkpoint inhibitors (ICIs) only benefit a subset of cancer patients, underlining the need for predictive biomarkers for patient selection. Given the limitations of tumor tissue availability, flow cytometry of peripheral blood mononuclear cells (PBMCs) is considered a noninvasive method for immune monitoring. This study explores the use of spectrum flow cytometry, which allows a more comprehensive analysis of a greater number of markers using fewer immune cells, to identify potential blood immune biomarkers and monitor ICI treatment in non-small-cell lung cancer (NSCLC) patients.

**Methods:**

PBMCs were collected from 14 non-small-cell lung cancer (NSCLC) patients before and after ICI treatment and 4 healthy human donors. Using spectrum flow cytometry, 24 immune cell markers were simultaneously monitored using only 1 million PBMCs. The results were also compared with those from clinical flow cytometry and bulk RNA sequencing analysis.

**Results:**

Our findings showed that the measurement of CD4+ and CD8+ T cells by spectrum flow cytometry matched well with those by clinical flow cytometry (Pearson R ranging from 0.75 to 0.95) and bulk RNA sequencing analysis (R=0.80, P=1.3 x 10-4). A lower frequency of CD4+ central memory cells before treatment was associated with a longer median progression-free survival (PFS) [Not reached (NR) vs. 5 months; hazard ratio (HR)=8.1, 95% confidence interval (CI) 1.5–42, P=0.01]. A higher frequency of CD4-CD8- double-negative (DN) T cells was associated with a longer PFS (NR vs. 4.45 months; HR=11.1, 95% CI 2.2–55.0, P=0.003). ICIs significantly changed the frequency of cytotoxic CD8+PD1+ T cells, DN T cells, CD16+CD56dim and CD16+CD56- natural killer (NK) cells, and CD14+HLDRhigh and CD11c+HLADR + monocytes. Of these immune cell subtypes, an increase in the frequency of CD16+CD56dim NK cells and CD14+HLADRhigh monocytes after treatment compared to before treatment were associated with a longer PFS (NR vs. 5 months, HR=5.4, 95% CI 1.1-25.7, P=0.03; 7.8 vs. 3.8 months, HR=5.7, 95% CI 169 1.0-31.7, P=0.04), respectively.

**Conclusion:**

Our preliminary findings suggest that the use of multicolor spectrum flow cytometry helps identify potential blood immune biomarkers for ICI treatment, which warrants further validation.

## 1 Introduction

The field of cancer immunotherapy has undergone a renaissance due to a greater understanding of the complex pathways that regulate tumor-induced immunosuppression. Immune checkpoint inhibitors (ICIs) targeting programmed cell death protein 1 (PD-1), its ligand PD-L1, and cytotoxic T-lymphocyte-associated protein 4 (CTLA-4) pathways have become the most potent and durable cancer immunotherapy, which has been shown to increase tumor control and extend life in many cancer types (1, 2). As ICIs have become an essential treatment modality in non-small cell lung cancer (NSCLC) treatment for those patients without EGFR or ALK mutations (3, 4), it becomes necessary to develop reliable methods of predicting and monitoring immune responses to these drugs (5, 6). Currently, the only validated biomarkers for ICI treatment are derived from tumor tissue: PD-L1 protein expression or tumor mutational burden (7). However, obtaining tumor biopsies may not be feasible in patients who are critically ill or whose tumors are in inaccessible locations.

Alternatively, peripheral blood offers a minimally invasive approach for examining tumor biology and tracking the evolution of molecular and immune biomarkers throughout cancer therapy (8, 9). We and others have shown that early changes in blood immune cells can correlate with clinical response to ICIs (10–12). However, the assessment for the phenotype changes of immune responses in lung cancer patients following ICI therapy has been hampered by limitations in the multiplexing capability of traditional flow cytometry, the high skill requirement for multiplexing a higher number of fluorochromes, and cost of equipment and filters for advanced multiplexing. Other options, such as mass cytometry (13) (e.g., CyToF), allow for significantly greater multiplexing of antigens, but are accompanied by higher costs and decreased sensitivity compared to conventional flow cytometry (14).

Traditional flow cytometry relies on matched filter and detector sets to measure the fluorescence emission peak for each fluorochrome marker. However, this method has limited resolution and multiplexing capabilities, which has driven the development of more sophisticated and sensitive instruments, often at increased costs when advanced multiplexing is required. In contrast, spectrum flow cytometry employs prisms to capture the complete emission spectrum across the entire visible light wavelength range for each fluorophore using an array of detectors.

Instead of using compensation to correct for fluorescence spillover like conventional flow cytometry, spectrum flow cytometry employs a technique known as spectrum unmixing. This process involves a mathematical algorithm that leverages the distinct spectrum signature of each fluorophore to differentiate multiple fluorophore signatures within a sample. Consequently, fluorophores with nearly identical peak emissions but differing off-peak emissions can be distinguished and utilized simultaneously in a panel (15, 16). As a result, spectrum flow cytometry can differentiate fluorophore combinations that conventional systems are unable to discern. This increased differentiation capability allows for enhanced flexibility when designing highly complex multicolor panels for comprehensive immunophenotypic analysis. Additionally, since more markers can be evaluated concurrently, spectrum flow cytometry effectively addresses the issue of limited sample availability by expanding the range of immune cell subtypes that can be assessed from a single sample. The aim of this study was to showcase the application of a 24-color spectrum flow cytometry panel in monitoring immune cell alterations in peripheral blood mononuclear cells (PBMCs) obtained from freshly collected or cryopreserved samples from lung cancer patients undergoing treatment with ICIs.

## 2 Materials and methods

### 2.1 Study patients and biospecimen collection

**Figure 1:**
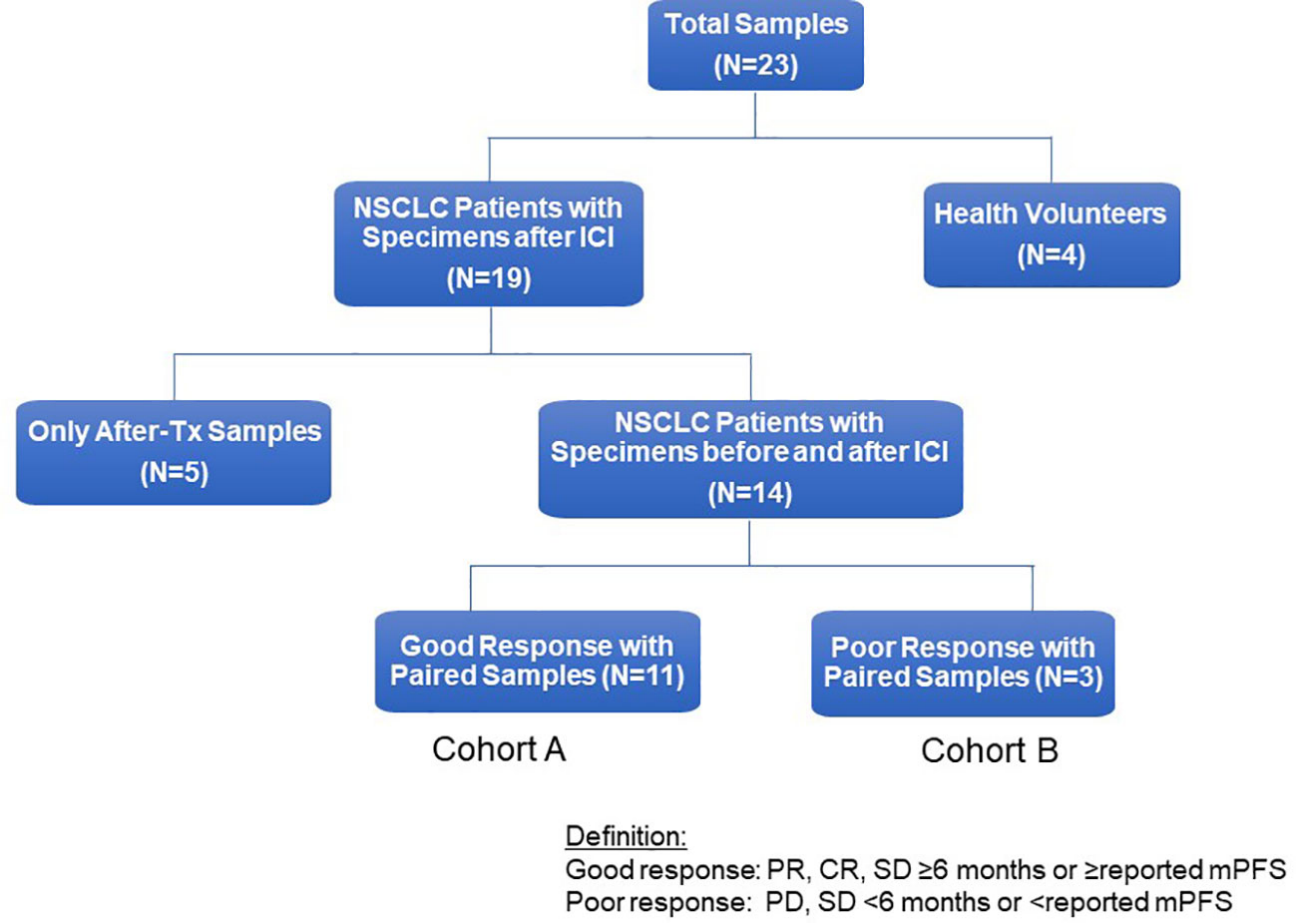
Flow chart of study patients. Flow chart for study patients. ICI, immune checkpoint inhibitor; NSCLC, non-small cell lung cancer; Tx, treatment, PD, progressive disease; PR, partial response; mPFS, median Progression-Free Survival.

Whole blood samples (30 mL) were collected in sodium-EDTA Vacutainer tubes (BD) from patients (N=19) and healthy human donors (N=4) *via* an Institutional Review Board (IRB) approved protocol (University of California, Davis Protocol No. 226210) (**Figure 1**). Demographics, immune and molecular biomarkers, blood collection, and treatment information of 19 study patients were abstracted from electronic medical records (**Table 1**). Of these 19 patients, 14 had blood samples before and after ICI treatment, and 5 only had post-treatment blood samples. Of the 14 patients with paired blood samples, 11 (78.5%) had good tumor response (cohort A), and 3 (22.5%) had poor tumor response (cohort B). PBMCs were isolated within 4 hours of collection at room temperature using the Ficoll-Paque Plus (GE Healthcare, Chicago, IL, USA) density gradient. A total of 2-5 x 10^7^ cells were cryopreserved in RPMI-1640 medium (Biowest, Nuaillé, France) with 10% dimethyl sulfoxide (DMSO, Sigma-Aldrich, St. Louis, MO, USA) and 10% heat-inactivated fetal bovine serum (FBS, Gibco™, ThermoFisher Scientific, Waltham, MA, USA) (17–19). The cell suspensions were aliquoted into 1 mL portions in freezing tubes and stored overnight at -80°C using a Cryo-Safe™ Cooler before being transferred to liquid nitrogen for long-term storage. To thaw, PBMCs were immersed in a 37°C water bath without agitation. Thawed PBMCs were then gradually added to 19 mL of pre-warmed complete RPMI1640 medium, supplemented with 10% FBS and 1% penicillin/streptomycin (Gibco), and gently mixed by inverting the tube. Following two washes with PBS, viable PBMCs were counted using trypan blue dye.

**Table 1:** Patient demographics, histology, biomarkers, blood collection, and treatment information of study patients.

Category	All Patients: N (%)	Paired Patients: N (%)
Blood collection timepoints	Post-treatment	Pre-treatment
No. Patient	19	14
Age: median (SD)	68.0. (8.9)	67.0 (9.3)
Gender: Female	8 (42%)	8 (57.1%)
Race/ethnicity:		
Non-Hispanic White	17 (89.5%)	14 (100%)
Other	2 (10.5%)	0 (0%)
Smoking history		
Yes	16 (84.2%)	13 (92.9%)
Stage		
II	2 (10.5%)	2 (14.3%)
III	1 (5.2%)	1 (7.1%)
IV	16 (84.3%)	11 (78.6%)
Histology		
LUAD	11 (57.9%)	8 (57.1%)
LUSC	7 (36.8%)	6 (42.9%)
Other	1 (5.3%)	0
PD-L1 expression		
Negative (0%)	5 (26.3%)	7 (50.0%)
Positive (≥1%)	12 (63.2%)	7 (50.0%)
NA	2 (10.5%)	NA
Drug choice		
*ICI Mono*	8 (42.1%)	4 (28.6%)
Pembrolizumab	7 (87.5%)	4 (100%)
Durvalumab	1 (12.5%)	0
***ICI Combo***	11 (57.9%)	10 (71.4%)
PD-1/PD-L1 + CTLA4 inhibitors or ant-LAG3 antibody	6 (54.5%)	5 (50.0%)
ICI +Chemotherapy	5 (45.5%)	5 (50.0%)

Multiple samples from the same patients were collected at various time points throughout their disease progression. Strict operating procedures were employed for sample collection, processing, and storage to reduce any potential variation in sample handling.

### 2.2 Flow cytometry staining

Fresh and thawed PBMCs were assessed for immunophenotypic alterations in key innate and adaptive immune cell populations using a 24-color antibody panel on the Aurora spectrum flow cytometer (Cytek Biosciences, Inc, California). The immune cell subtypes examined included various T cell subsets (such as effector, activated, memory, exhausted, and regulatory subsets), B cells, and natural killer (NK) cells. Specifically, 1x10^6^ PBMCs were stained with well-established antibodies targeting markers of interest, including CD3, CD45, CD4, CD8, PD-1, CD25, CCR7, CD127, CD11b, HLA-DR, CD11c, CD56, CD16, and others, following standard protocols. Central Memory (CM) T cells are defined as CCR7+ and CD45RA- subsets of CD4+ or CD8+ T cells. **Supplemental Table S2** summarizes the antibodies and dyes used for multicolor flow cytometry in this study. The immune cell subtypes analyzed by this multicolor flow cytometry panel are illustrated in **Supplemental Figure S1**. **Supplemental Figure S2** illustrates the gating strategy for a 24-marker panel and immune cell subtypes of the multicolor spectrum flow cytometry. Immuno-labeling of cells was performed by adding 150 μL Zombie Near IR fixable viability cell dye (Biolegend, San Diego, CA, USA) at a dilution of 1:2500 in PBS and left on ice to incubate for 20 minutes in the dark. One million PBMCs in 100 μL of PBS + 2% FBS were added to each DURAClone tube (Beckman Coulter Inc, CA). Cells were stained in 100 μL predetermined antibody volumes. Tubes were vortexed for 10 seconds and incubated for 15 minutes in the dark. To remove unbound antibodies prior to fixing, 300 µL of PBS with 2% FBS was added to each tube, cells were spun down at 800 x *g* for 5 minutes, and the supernatant was discarded. Cells were resuspended in staining buffer [PBS with 1% bovine serum albumin and 0.1% sodium azide (Sigma-Aldrich)] and acquired in the flow cytometer. We compared the expression of CD4+ and CD8+ cells by the spectrum flow cytometry with those of clinical flow cytometry at our institution.

### 2.3 Data acquisition, collection and statistical analysis

One-peak Rainbow Beads (Biolegend) informed voltage settings, and compensation controls were established using auto-compensation with single-color control ultra-comp beads (ThermoFisher Scientific). Voltages for each parameter minimized spillover between fluorophores. Data acquisition was performed on the Aurora spectrum flow cytometer (Cytek Biosciences, CA), and the SpectroFlo™ software was used for analysis. The resulting FCS files were examined using the Cytobank platform (Beckman Coulter Inc, CA) (20). Qualitative variables were summarized by frequency and percentage, while quantitative variables were reported as mean ± standard deviation (SD), unless stated otherwise. The 95% confidence interval for survival was calculated using the exact binomial distribution. Cell proportions or frequencies were presented as mean and SD for before and after ICI treatment cohorts and response subgroups. To compare two groups, Wilcoxon rank-sum tests were used (or Kruskal-Wallis tests for three cohorts). A two-sided P<0.05 indicated statistical significance. Due to the study’s exploratory nature, no statistical adjustment for multiple hypothesis testing was performed for multiple blood cell types (21). Statistical analyses were conducted using SAS version 9.4 (SAS Institute, Cary, NC). The best responses to systemic therapies, including complete or partial response (CR or PR), stable disease (SD), or progressive disease (PD), were evaluated using Response Evaluation Criteria in Solid Tumors (RECIST) version 1.1 (22). Progression-free survival (PFS) was calculated from the first cancer therapy administration to progression as defined by RECIST1.1 or death from any cause. Patients without progression at the time of analysis were censored at the initiation of new therapy or last follow-up. Good clinical response was determined in patients who achieved CR, PR, SD ≥6 months, or PFS exceeding the reported median PFS for each ICI therapy. Poor clinical response was defined in patients who had PD, SD <6 months, or PFS shorter than the reported median PFS for each ICI therapy. Overall survival (OS) was measured from the first administration of cancer therapy to death from any cause. Patients who were alive at the time of analysis were censored at the initiation of new therapy or last follow-up. Survival data were estimated using the Kaplan-Meier method and compared using the log-rank test in each cohort and response subgroups.

### 2.4 RNA sequencing analysis and estimation of T cell frequency

RNA was isolated from 1 million PBMCs using the RNeasy mini kit (Qiagen). RNA-sequencing was implemented at Novogene Corporation Inc. (https://en.novogene.com), which performed the quality control analysis and constructed the library using TruSeq Stranded Total RNA Sample Prep Kit (Illumina). Sequencing was performed using the NovaSeq 6000 system (NovaSeq PE150, Novogene UC Davis Sequencing Center). The sequencing data were accessible at the Gene Expression Omnibus (GEO) repository (accession number GSE235048) for analysis. Reads were aligned to human hg38 genome using Salmon with standard settings (23). Transcripts per million (TPM) counts for all genes were then inputted into CIBERSORTx (https://cibersortx.stanford.edu) for imputing cell fractions using the LM22 signature matrix, disabling quantile normalization. CD4+ T cell frequency was calculated by summing all the CD4+ subtypes (CD4+ naïve cells, CD4+ memory resting cells, CD4+ memory activated cells, follicular helper T cells, regulatory T cells). CD8+ T cell frequency was calculated by CIBERSORTx as pan-CD8+ T cell value.

## 3 Results

### 3.1 Validation of multi-color spectrum flow cytometry data by clinical flow cytometry and RNA sequencing

CD3+CD4+ and CD3+CD8+ cell frequencies are two commonly used parameters in the clinical flow cytometry analysis. Next, we compared the expression of CD3+CD4+ and CD3+CD8+ cell frequenciesby 24-color spectrum flow cytometry with those cell frequencies by clinical reports of classical flow cytometry at our institution. According to Pearson’s correlation analysis, there is a strong positive correlation between 24-color spectrum flow panels and clinical flow cytometry in detecting CD4+ and CD8+ populations in patient samples before and after treatment, ranging from 0.75 to 0.95 (**Supplemental Figures S3A–D**). As an orthogonal validation, RNA was isolated from the same PBMC samples, sequenced, and cell composition estimated by CIBERSORTx (23). Given that the standard CIBERSORTx cell types are different from the specific cell subtypes profiled by 24-color spectrum flow cytometry, we focused only on comparing the pan-CD4+ and pan-CD8+ T cell (CD3+) populations. We found a strong and significant correlation between the CIBERSORTx estimates and 24-color spectrum flow measurements for CD4+ T cells (r = 0.80, *p* = 1.3 x 10^-4^) and CD8+ T cells (r = 0.80, *p* = 1.3 x 10^-4^) (**Supplemental Figures S3E, F**).

### 3.2 Baseline level of immune cell subtypes in NSCLC patients

**Figure 2:**
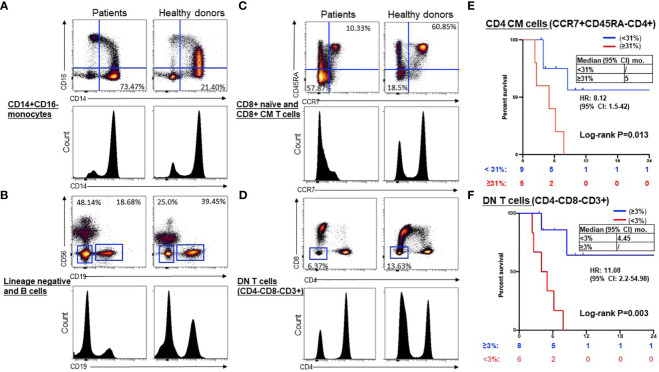
Representative dot plots and histograms of significantly different levels and prognostic significance in NSCLC patients compared to healthy volunteers at baseline. Representative dot plots and histograms illustrate significantly different levels of CD14+CD16- monocytes **(A)**, lineage negative and B cells **(B)**, CD8+ naïve and CD8+ CM T cells **(C)**, and DN (CD4-CD8-CD3+) T cells **(D)** in NSCLC patients compared to healthy volunteers at baseline. Kaplan-Meier curves showed low CD4+ CM (CCR7+ CD45RA-) T cells (< 31%, blue) were associated with longer PFS compared to high (≥31%, red) **(E)**, and higher DN (CD4-CD8-CD3+) T cells (≥3%, blue) were associated with longer PFS compared to low (< 3%, red) **(F)**. Groups were compared by the paired t-test. *P < 0.05 for statistical significance. CM, central memory; ICI, immune checkpoint inhibitors.

We compared the immune cell subtypes in PBMCs from NSCLC patients with those from healthy donors as the control group (**Table S2**). At baseline, CD8+ naive T cells, double-negative (DN) CD3+CD4-CD8- T cells, and B cells were significantly lower in the patient’s samples, whereas the CD14+CD16- monocytes, CD8+ CM cells, and lineage-negative cells were higher in patient’s samples than the control group (**Table 2**). Representative dot plots and histograms illustrate significantly different levels of CD14+CD16- monocytes (**Figure 2A**), lineage negative and B cells (**Figure 2B**), CD8+ naïve and CD8+ CM T cells (**Figure 2C**), and DN (CD4-CD8-CD3+) T cells (**Figure 2D**) in NSCLC patients compared to healthy volunteers at baseline. Of these immune cell subtypes, patients with a baseline CD4+ CM (CCR7+CD45RA-CD4+) T cell proportion of <31% exhibited longer median PFS compared to those with a proportion of ≥31% [not reached (NR) vs. 5 months; hazard ratio (HR) 8.1, 95% confidence interval (CI) 1.5–42, P = 0.01] (**Figure 2E**). Additionally, patients with a baseline double-negative (DN) (CD4-CD8-CD3+) T cell proportion of ≥3% demonstrated significantly improved PFS compared to those with a DN proportion of <3% (NR vs. 4.45 months; HR=11.1, 95% CI 2.2–55, P < 0.01) (**Figure 2F**). An optimal cut-off value for predicting PFS based on a receiver operating characteristic (ROC) curve was also examined for CD4+ CM T cells and DN T cells (**Supplementary Figures S4A, B**), respectively.

**Table 2:** Significantly changed immune cell subtypes before treatment.

Cell Marker	Patients (N=14)		Normal control (N=4)		P value
	Percent (%)	SD (%)	Percent (%)	SD (%)	
CD14+CD16-	62.7	25.6	24.0	10.5	0.02
CD8 naive	9.5	8.3	56.2	23.2	0.001
CD8 CM	31.7	14.4	16.3	6.1	0.03
LINEAGE NEGATIVE	48.0	26.8	20.6	3.2	0.03
DN	6.5	7.8	13.3	1.6	0.048
B cells	21.9	14.1	40.6	10.1	0.03

### 3.3 Blood immune cell changes after ICI treatment in NSCLC patients

**Figure 3:**
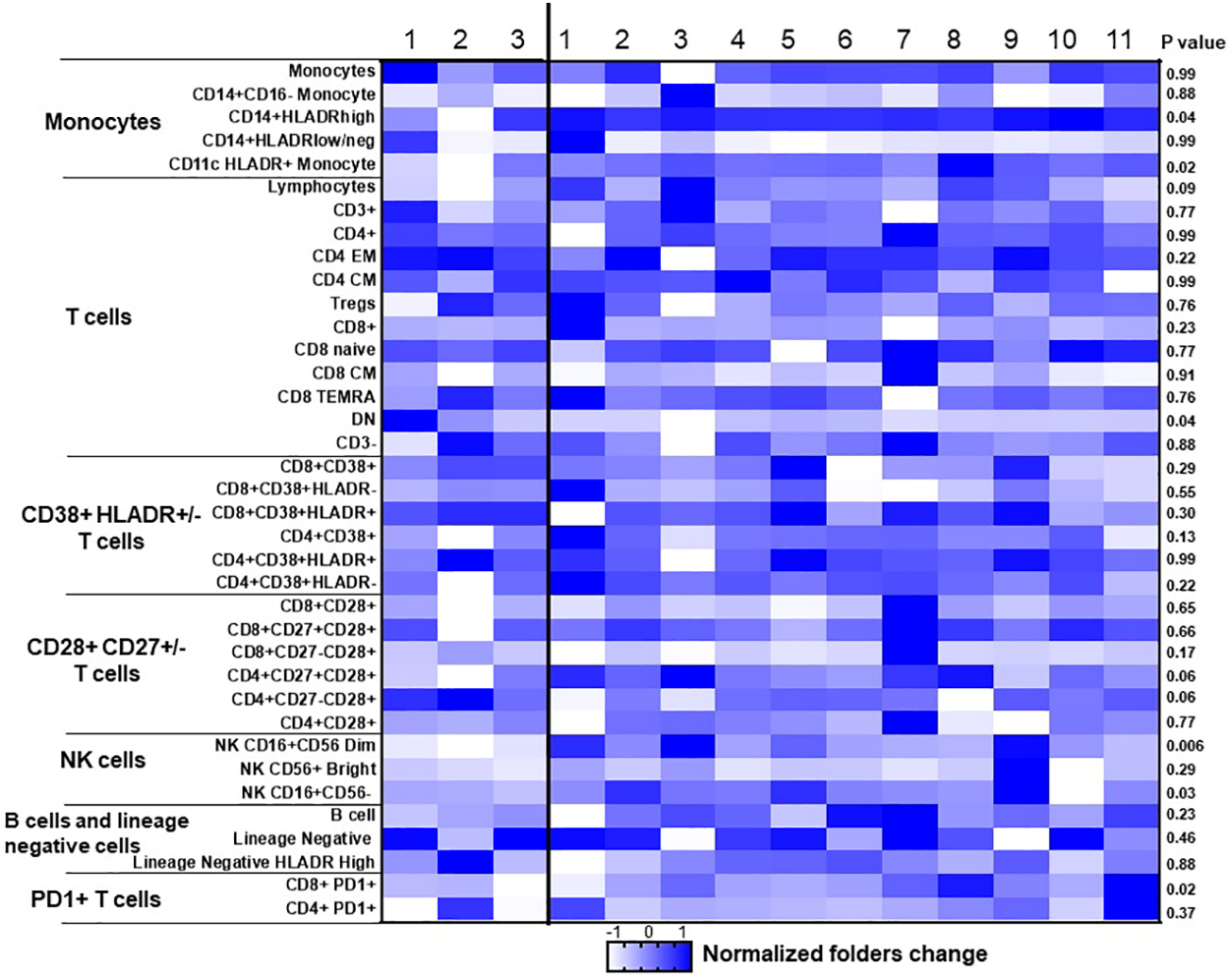
Dynamic changes of immune cells before and after ICI treatment in NSCLC patient samples. Heat map of 37 cell populations defined from flow cytometric analysis of a subset of NSCLC samples from responders (*n* = 11) and nonresponders (progressive disease; *n* = 3). For each population, two-tailed *P* values were calculated using the Wilcoxon rank-sum test. Data for each row were logged and mean-centered; each column shows data from one sample. *P < 0.05 for statistical significance. EM, effector memory; CM, Central memory; NK, Natural killer; DN, double negative.

**Figure 4:**
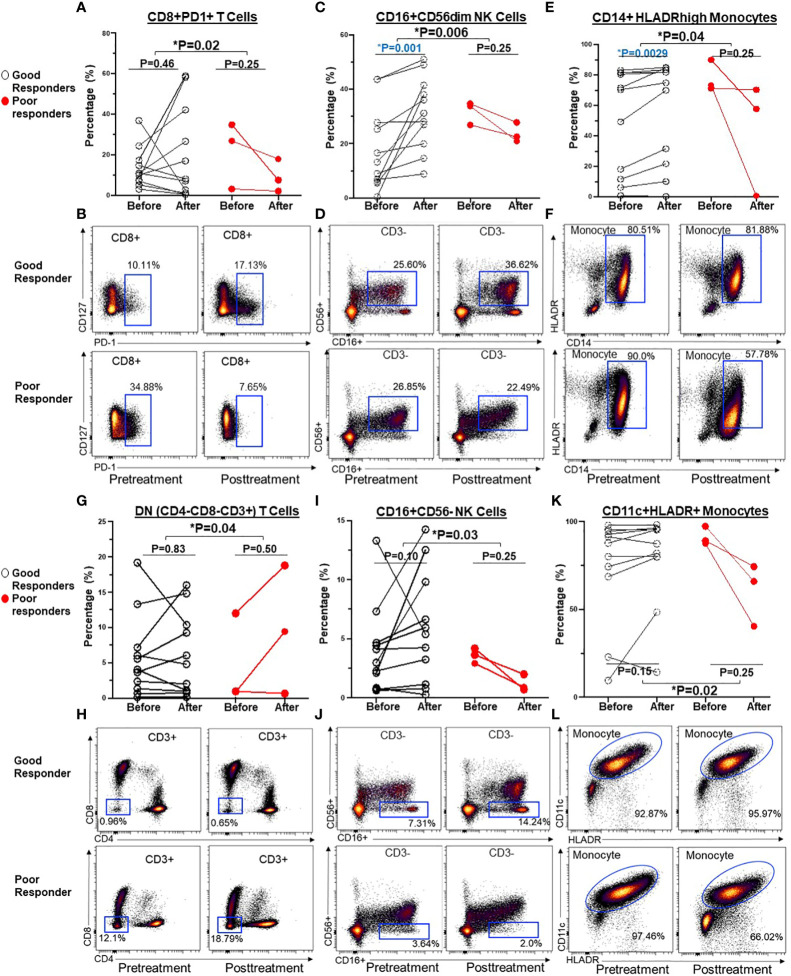
Before and after graphs for dynamic changes of immune cells after ICI treatment were associated with clinical benefits in NSCLC patients. Compared to poor responders, ICI treatment led to significant alterations in the frequencies of CD8+ PD-1+T cells **(A, B)**, CD16+CD56dim NK cell **(C, D)**, CD14+HLADRhigh monocyte **(E, F)**, CD4-CD8- DN T cells **(G, H)**, CD16+CD56- NK cells **(I, J)**, and CD11c+ HLADR+ monocytes **(K, L)** in overall good responders. Groups were compared by the paired t-test. *P < 0.05 for statistical significance. Blue highlights statistically significant difference before and after ICI treatment.

To assess the impact of ICI therapy on immune cells, we analyzed T cells, B cells, NK cells, and monocytes, along with their subtypes, in the peripheral blood of patients with advanced NSCLC, before and after ICI treatment (**Table S3**). Individual NSCLC patients had unique patterns of immune cell expression and changes to ICI treatment (**Figure 3**). Compared to poor responders, ICI treatment led to significant alterations in the frequencies of CD8+PD-1+ T cells (P=0.02, **Figures 4A, B**), CD16+CD56dim NK cells (P=0.006, **Figures 4C, D**), CD14+HLADRhigh monocytes (P=0.04, **Figures 4E, F**), CD4-CD8- DN T cells (P=0.04, **Figures 4G, H**), CD16+CD56- NK cells (P=0.03, **Figures 4I, J**), and CD11c+ HLADR+ monocytes (P=0.02, **Figures 4K, L**) in overall good responders. Although all 3 poor responders had decreased CD8+PD1+ T cells, 6 out of 11 (54.5%) good responders also had decreased CD8+PD1+ T cells (**Figure 4A**). In addition, we observed a significant increase in both CD16+CD56dim NK cell frequency and CD14+HLADRhigh monocyte frequency among good responders after ICI therapy, which was not seen in poor responders (**Figures 4B, E**, respectively). Nonetheless, there were no significant disparities in the frequencies of CD4+ T cells, CD8+ T cells, CD19+ B cells, CD38+ T cells, and CD27+ T cells (**Table S3**; **Figure 3**).

### 3.4 Dynamic changes of CD16+CD56dim NK cells and CD14+HLADRhigh monocytes were associated with clinical benefits in NSCLC patients

**Figure 5:**
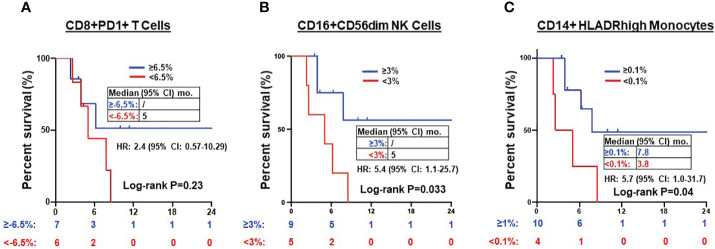
Prognosis of after treatment immune cells in NSCLC patients. Kaplan-Meier PFS estimates according to post-treatment changes (post-treatment minus baseline) in CD8+PD1+ T cells **(A)**, CD16+CD56dim NK cells **(B)** and CD14+HLADRhigh monocytes **(C)**. Tick marks indicate censored data. Groups were compared using the log-rank test. *P* < 0.05 for statistical significance.

Next, we explored whether the expression of these T cell, NK cell, and monocyte subtypes before and after ICI treatment correlated with clinical outcomes. There was no significant prognostic correlation in the expression of PD1+CD8+ T cells (PFS NR vs. 5 months, HR=2.4, 95% CI 0.57-10.29, P=0.23) (**Figure 5A**) nor DN T cells (*data not shown*). However, a post-treatment increase in CD16+CD56dim (but not CD16+CD56- NK cell) frequency of ≥3% following ICI treatment was associated with superior PFS (NR vs. 5 months, HR=5.4, 95% CI 1.1-25.7, P=0.033) (**Figure 5B**). Of the monocytes, an increase in CD14+HLADRhigh monocyte frequency by ≥0.1% following ICI treatment was associated with improved PFS (7.8 vs. 3.8 months, HR=5.7, 95% CI 1.0-31.7, P =0.04) (**Figure 5C**).

### 3.5 Serial assessments of immunophenotypic changes of PBMCs in patients who received ICI combination by 24-color spectrum flow cytometry

**Figure 6:**
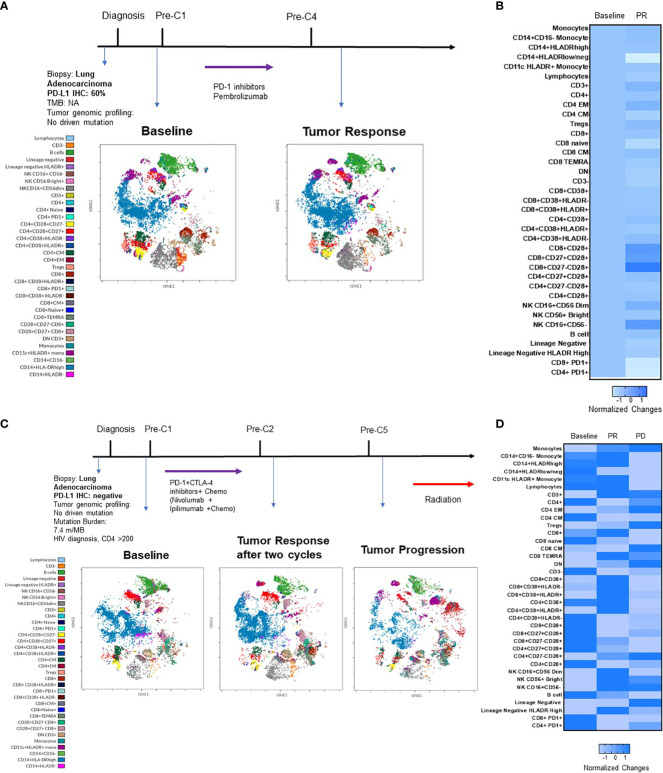
Immunophenotypic changes of PBMCs in two patients who received ICI alone or in combination. **(A)** summarizes the key events in the clinical course of a patient with good response to pembrolizumab. Cell amounts before and after ICI treatment are represented by a t-distributed stochastic neighbor embedding (t-SNE) plot displayed as a 2D plot using the resultant t-SNE 1 and t-SNE 2 dimensions according to the per cell expression of the 24 proteins assayed. Colors represented each defined cellular subtype as indicated. **(B)** Heatmap representing the fold-change in percentage of each cellular subtype before and after treatment. **(C)** summarizes the clinical course of a patient with good response to pembrolizumab and then progression in a similar t-SNE plot and heatmap **(D)**.

Quantitative assessment of immune biomarkers on PBMCs during disease course was assessed using this multicolor panel by spectrum flow cytometry. **Figure 6** summarizes the clinical course of two patients who received ICIs or ICI and chemotherapy combination at diagnosis. Case 1 (**Figures 6A, B**) was a 93-year-old female, former smoker who was diagnosed with stage 4 NSCLC adenocarcinoma in August 2020. The tumor had high PD-L1 IHC expression (tumor proportion score was 60%) (DAKO, clone 22C3). Tumor genomic profiling did not identify any actionable oncogene alterations. The patient received pembrolizumab for four cycles with significant clinical and radiographic improvements. As shown in viSNE land (**Figure 6A**) and heatmap (**Figure 6B**), the ICI treatment increased CD3+ T cells, CD16+CD56dim NK cells, CD16+CD56- NK cells, CD4+ Effector Memory (EM), CD8+ CM T cells, CD14+HLADRhigh monocyte and decreased CD4+ CM, Double Negative (DN), CD8+ T cells, lineage negative cells, CD14+HLADRlow/neg monocytes, PD1+ CD4+ cells, and PD1+ CD8+ cells. The patient continued to have pembrolizumab for 35 cycles with clinical remission. The second case (**Figures 6C, D**) was a 66-year-old male with controlled AIDS (CD4 >200 cells per cubic millimeter on antiviral treatment) for over ten years. The patient was diagnosed with stage 4 NSCLC adenocarcinoma in June 2020. The tumor was negative for PD-L1 IHC stain with a tumor mutation burden of 7.4 m/MB. He received PD-1 and CTLA-4 inhibitors and chemotherapy for two cycles with a partial response. The patient subsequently received IO combination therapy for three cycles before tumor progression. As shown in viSNE analysis (**Figure 6C**) and heatmap (**Figures 6D**), the ICI treatment increased CD3+, NK cells, CD4+ EM, and decreased CD4+ CM, DN CD3+ cells, naive cells, lineage negative cells, CD14+HLADRhigh monocytes, PD1+CD4+ T cells, and PD1+CD8+ T cells when the tumor responded to the therapy. However, CD3+, CD16+CD56dim NK cells, CD14+HLADRhigh monocytes, and CD8+ cells were found to decrease when the patient experienced the tumor progression.

## 4 Discussion

Although the tumor-specific cytotoxic T cells are the prime targets of ICIs, they can modulate the expression of additional immune cells and molecules in the tumor microenvironment (TME) and lymphoid organ frequencies (24, 25). These immune cell subtypes include macrophages, T cells, NK cells, monocytes, B cells, dendritic cells, myeloid-derived suppressor cells (MDSCs), and neutrophils. These components can collectively contribute to the regulation of tumor cell immune evasion. Moreover, imbalances in the levels of specific immunoregulatory cells and cytokines within the TME may influence tumor responsiveness (26). Cancer cells may employ several immune escape mechanisms, including inadequate presentation of tumor neoantigens, secretion of inhibitory chemokines or cytokines (e.g., TGF-β, IL-10), mutations that make molecules unrecognizable by the immune system, and recruitment of suppressive cells (e.g., Treg, MDSC) (27). A growing body of evidence suggests circulating immune cells in peripheral blood of patients may reflect the dynamic immune cell changes in the tumor tissue (28).

Our study presents several important clinical implications through the immunophenotypic analysis of immune subtypes in patient PBMCs before and after ICI treatment by the multicolor spectrum flow cytometry. Firstly, we found that at baseline monocytes, CD8+ naive T cells, double-negative CD3+CD4-CD8- T cells, and B cells were significantly lower in the patient’s samples, whereas the CD14+CD16- monocytes, CD8+ CM cells, and lineage-negative cells were higher in patient’s samples compared to healthy donors. Of these markers, NSCLC patients with a lower baseline proportion of CD4+ CM cells (<31%) or a higher baseline proportion of DN (CD4-CD8-CD3+) T cells (≥3%) experienced longer median PFS to ICI treatment. The function of CD4+ helper T cells has been shown to be crucial for effective CD8+ T cell responses to ICI therapy. CD4+ T cells can promote tumor regression through various mechanisms, including cytokine secretion (IL-2), enhancing tumor-specific CD8+ T-cell function, or directly eliminating cancer cells (29). Several preclinical studies have demonstrated that tumor-specific CD4+ T cells might recognize immunogenic mutations (30, 31). Consistent with our result, Tada et al. showed that a decrease in baseline CD4+ CM T cells in 7 out of 10 patients with head and neck squamous cell carcinoma exhibiting PR or SD to PD-1 inhibitor nivolumab (32). However, due to the small sample size, the result did not achieve statistical significance. Furthermore, the expansion of CD4+ naïve T cells to CD4+ CM T cells after ICI therapy predicted long-term survival benefits in patients with malignant melanoma (33). CD4+ CM T cells, characterized by CCR7 or CD62L expression and lacking CD45RA, can circulate within secondary lymphoid organs. While the functional interplay of CD4+ memory cells directly shapes the effects of PD-L1/PD-1 inhibitors on CD8+ anti-tumor responses, the exact mechanisms mediating this response remain elusive (29). Additionally, various studies have shown other T cell subtypes involved in response to ICI therapy. For instance, a significant decrease of CD4+ FOXP3- PD-1 high T cells during the initial stages of therapy correlated with an improvement in OS (30). There was a trend suggesting that a lower proportion of circulating CD4+ T cells, including CD38+CD4+ T cells in peripheral blood, could indicate a favorable prognosis (31). Further studies are needed to characterize the role of different T cell subtypes in mediating response to ICIs.

Secondly, we observed that individual NSCLC patients had unique patterns of blood immune cell expression and changes to ICI treatment (**Figure 3**). Although all 3 poor responders had decreased frequencies of CD8+PD1+ T cells, 6 out of 11 (54.5%) good responders also had decreased frequencies of CD8+PD1+ T cells (**Figure 4A**). Overall, there was a correlation between increased frequencies of cytotoxic CD8+PD1+ T cells in PBMCs and good clinical responses to ICIs. This finding aligns with previous reports that PD-1 expression in CD8+ tumor infiltrating lymphocytes (TILs) within tumor samples had clonally expanded tumor-reactive lymphocytes (34, 35). A majority of patients experiencing clinical benefits demonstrated CD8+ PD-1+ T-cell responses within four weeks of therapy (36). We further showed that ICI treatment modulated the expression of subtypes of NK cells and monocytes. Although there was no statistically significant difference in the expression of NK cell subtypes in NSCLC patients before ICI treatment compared to healthy donors (**Table S2**), we observed ICI treatment led to significant increase in the frequencies of CD16+CD56dim NK cell (P=0.006) (**Figures 4C, D**) and CD16+CD56- NK cells (P=0.03) (**Figures 4I, J**) compared to poor responders. Furthermore, an increase in the CD16+CD56dim NK cell frequency of ≥3% after ICI treatment compared to before treatment was associated with a longer PFS (NR vs. 5 months, HR=5.4, 95% CI 1.1-25.7, P=0.03) (**Figure 5B**). Our findings are consistent with several reports showing that NK cells, either alone or in combination with cytotoxic T cells, play an essential role in mediating tumor response to ICI treatment in multiple cancer types (32, 33, 37). In mouse models, NK cells promote the function of cytotoxic T cells in response to anti-PD-L1 treatment (38, 39). NK cells also acted as the primary cytotoxic cells in tumors with low MHC expression, even in PD-L1 negative tumors (40). A single dose of tumor vaccine targeting resistant tumors by dual T and NK cells was able to increase CD8+ and CD4+ T cell frequencies by 17.9 and 29.3 fold, and NK cell counts by about 40 fold, respectively, compared to the control in murine models (41). Recently, the expression of NK cell frequency in the peripheral blood was independently associated with longer survival of gastric cancer patients and colorectal patients (33, 37). The frequency of NK cells was positively correlated to the frequencies of T and B lymphocytes (33). However, these reports did not analyze the CD16 and CD56 subtypes of NK cells. In this study, we found that CD16+CD56dim NK cells played a more significant role in mediating response to ICI compared to CD16+CD56- NK cells. CD16+CD56dim NK cells comprise the majority of circulating human NK cells, which are the most cytotoxic NK cells. Upon target recognition, CD16+CD56dim NK cells release perforin and granzyme granules and mediate antibody-dependent cellular cytotoxicity through CD16 (FcɣRIII) to clear cancer cells. In contrast, increased CD16+CD56- NK cells were associated with immune escape from innate immunity during AML progression (42).

Previous studies suggested that CD14+HLADRhigh, low or neg monocytes play distinct roles in the regulation of inflammatory and immune-suppressive conditions (43, 44). Patients who responded to ipilimumab had significantly lower levels of pre-treatment CD14+HLA-DRlo/neg monocytes compared to their non-responsive counterparts (45). Another independent study linked lower pre-treatment frequencies of CD14+HLA-DRlo/neg monocytes to improved OS of patients (46). In this study, we did not observe any significant difference in the pre-treatment frequency of CD14+HLADRhigh and low/neg monocytes in NSCLC patients compared to healthy donors (**Table S2**). Instead, we found that there were significant increases in the frequency of CD14+HLADRhigh monocyte (P=0.04) (**Figures 4E, F**), and CD11c+HLADR+ monocytes (P=0.02) (**Figures 4K, L**) in good responders compared to poor responders after ICI treatment. Of these two monocyte subtypes, only an increase in CD14+HLADRhigh monocyte frequency by ≥0.1% following ICI treatment was associated with improved PFS (7.8 vs. 3.8 months, HR=5.7, 95% CI 1.0-31.7, P=0.04) (**Figure 5C**). Consistent with our results, single cell analysis revealed that high levels of CD14+CD16- HLADRhigh monocytes before ICI correlated with significantly increased PFS in melanoma patients (47) and NSCLC patients (48). Further study is warranted to validate these findings in NSCLC patients and delineate underlying mechanisms.

Individual NSCLC patients had unique patterns of immune cell expression and changes to ICI treatment and other cancer therapy (**Figure 6**). The 24-color spectrum flow cytometry assay may be used to monitor treatment response during the disease course with the optimal goal of improving the prediction of patient responses to cancer therapy and identify those who may benefit most from a specific treatment. However, this study has several limitations, including a small sample size, a retrospective design, and no adjustment for multiplicity due to its exploratory nature. Additionally, we did not analyze the changes in T cell receptor repertoire and various immunoregulatory cytokines in the blood. Potential selection bias and imbalances in patients’ baseline characteristics and treatment history could have influenced the outcomes. Compared to the liquid biopsy for tumor genomic profiling of plasma circulating tumor DNA, flow cytometry of blood immune cells requires special skills and the cost of appropriately collecting and processing blood immune cells in a timely manner. Before this method can be used in routine practice, the sensitivity, turnaround time, and cost need to be evaluated. Further study is warranted to use the established multicolor flow cytometry and RNA sequencing tools to monitor the dynamic changes of blood immune cells during cancer immunotherapy treatment.

## 5 Conclusions

Multicolor spectrum flow cytometry can simultaneously evaluate 1 million blood immune cells for changes in major immune cell subpopulations in NSCLC patients receiving ICIs. Our data support the critical role of subsets of T cells, NK cells and monocytes in mediating response to ICI. Further studies are needed to validate the predictive biomarkers and assays to select the appropriate patients for ICI therapy.

## Data availability statement

The RNA sequencing data presented in the study are deposited in the Gene Expression Omnibus (GEO) repository, accession number GSE235048. Other original contributions presented in the study are included in the article/**Supplementary Material**. Further inquiries can be directed to the corresponding author.

## Ethics statement

The studies involving human participants were reviewed and approved by University of California, Davis Institutional review board (IRB)-approved protocol (Protocol No. 226210). The patients/participants provided their written informed consent to participate in this study.

## Author contributions

TL contributed to the conception and design of the study. WM, SW, and TL contributed to the patient sample and data collection. WM, SW, SL, ET, BM, MJ, DM, VV, JC, QZ, JVD, SC, and TL contributed to the acquisition, analysis, or interpretation of data. WM, SW, SL, DM, SC, and TL drafted and revised the manuscript. All authors contributed to the article and approved the submitted version.
