# Supplementary material for: Dynamic evaluation of blood immune cells predictive of response to immune checkpoint inhibitors in NSCLC by multicolor spectrum flow cytometry
Source: Front Immunol. 2023 Aug 10;14:1206631. doi: 10.3389/fimmu.2023.1206631 (PMC10449448; doi:10.3389/fimmu.2023.1206631)
Supplement: Supplementary file 1 [file DataSheet_1.pdf]

Supplemental Figure S1. Summary of Immune Cell Subtypes by the 24-color spectral flow cytometry panel

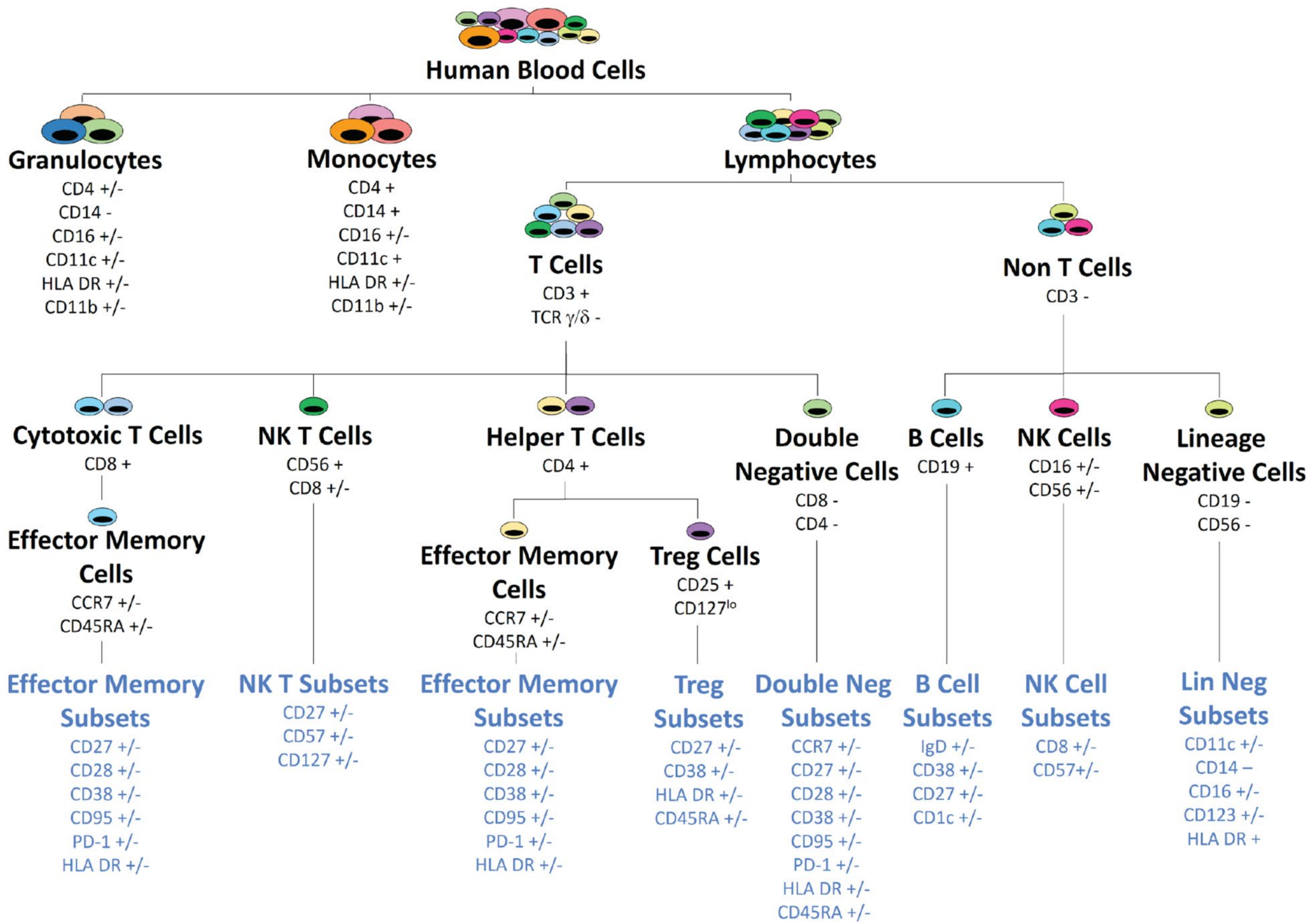

Supplemental Figure S2. Representative data showing the gating strategy for each panel and populations listed in Supplemental Table 1.

(A)

- Ungated
  - Singlets
    - Live
      - Lymphocytes
        - CD3-
          - B cell
          - Lineage negative
            - Lineage negative HLADR high
          - NK Cell
            - CD16+ CD56 dim NK cell
            - CD16+ CD56- NK cell
            - CD56 bright NK cell
        - CD3+
          - CD4+
            - CD4+Naive
            - CD4+CM
            - CD4+EM
            - CD4+CD28+CD27+
            - CD4+CD28+CD27-
            - CD4+CD38+HLADR high
            - CD4+CD38+HLADR low/neg
            - CD4+PD-1
            - Treg
          - CD8+
            - CD8+Naive
            - CD8+CM
            - CD8+TEMRA
            - CD8+CD28+CD27+
            - CD8+CD28+CD27-
            - CD8+CD38+HLADR high
            - CD8+CD38+HLADR low/neg
            - CD8+PD-1
          - Double negative (DN)
      - Monocytes
        - CD14+CD16-
        - CD14+CD16+
        - CD14+CD16+ nonclassical
        - CD14+HLADR high
        - CD14+HLADR low/neg
        - CD11c+HLADR+

(B)

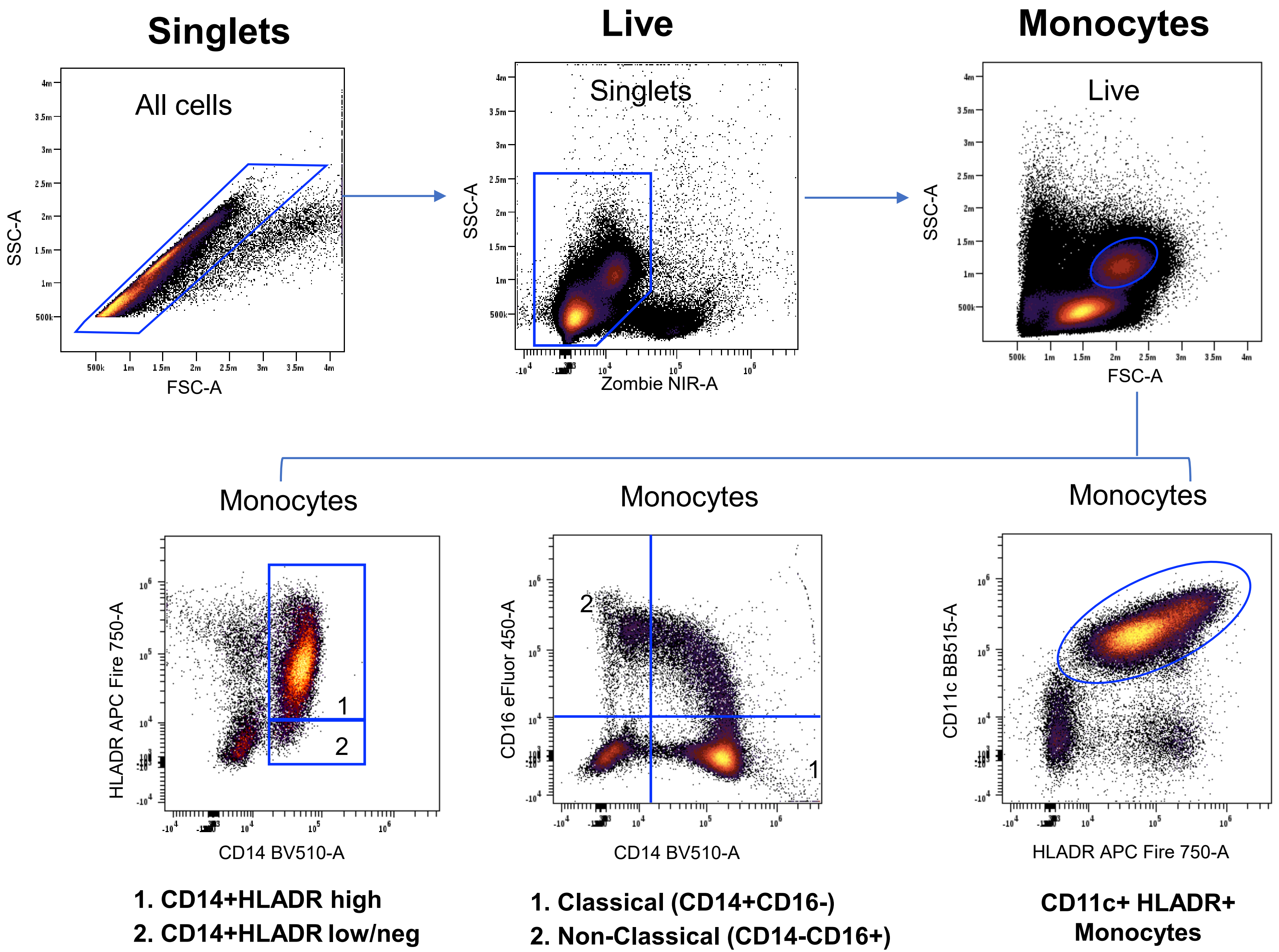

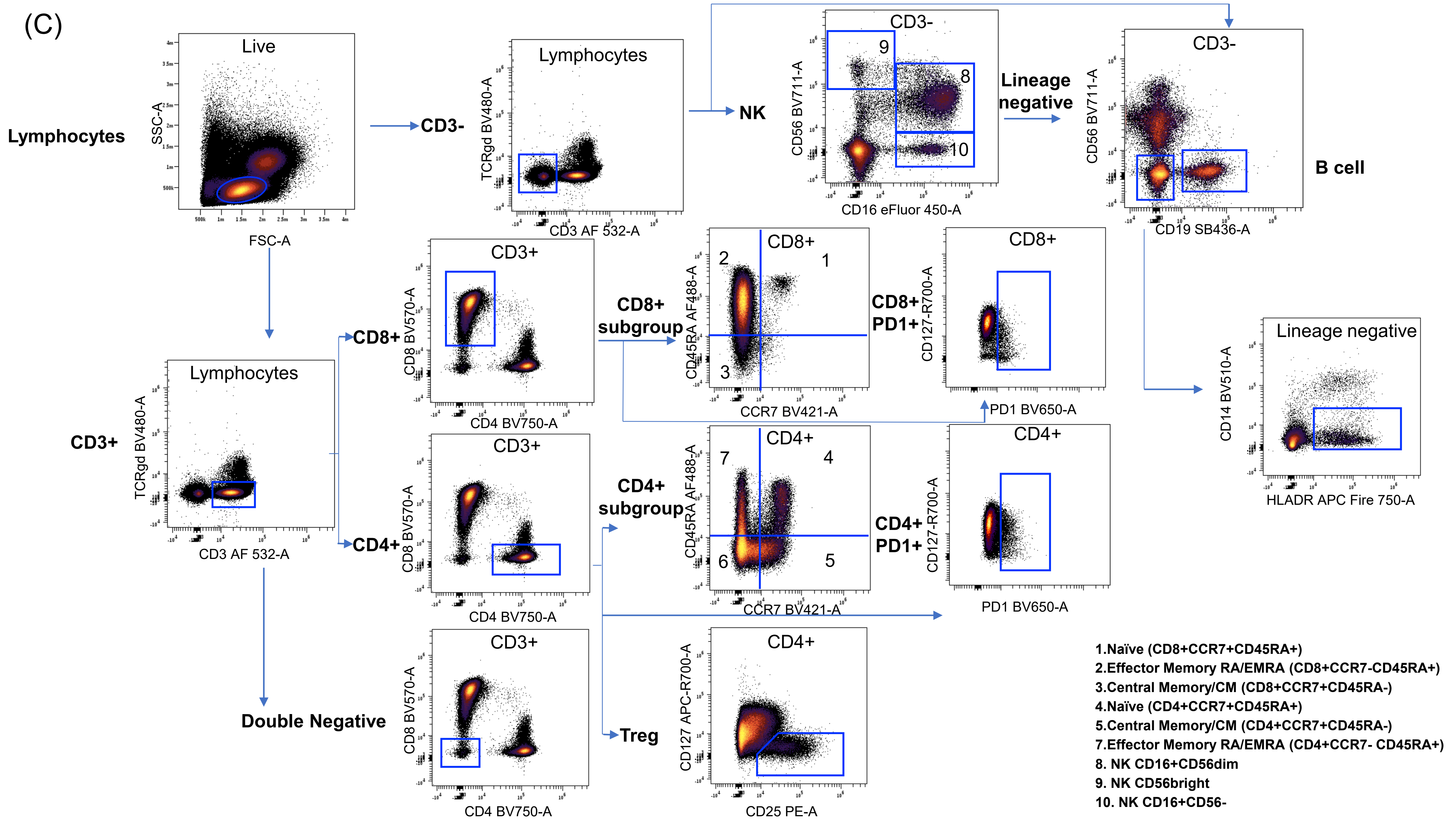

(C)
Lymphocytes
Live
SSC-A
FSC-A
CD3-
Lymphocytes
TCRgd BV480-A
CD3 AF 532-A
NK
CD3-
CD56 BV711-A
CD16 eFluor 450-A
9
8
10
Lineage negative
CD3-
CD56 BV711-A
CD19 SB436-A
B cell
CD3+
Lymphocytes
TCRgd BV480-A
CD3 AF 532-A
CD8+
CD3+
CD8 BV570-A
CD4 BV750-A
CD8+ subgroup
CD8+
CD45RA AF488-A
CCR7 BV421-A
2
1
3
CD8+ PD1+
CD8+
CD127-R700-A
PD1 BV650-A
Lineage negative
CD14 BV510-A
HLADR APC Fire 750-A
CD4+
CD3+
CD8 BV570-A
CD4 BV750-A
CD4+ subgroup
CD4+
CD45RA AF488-A
CCR7 BV421-A
7
4
6
5
CD4+ PD1+
CD4+
CD127-R700-A
PD1 BV650-A
Double Negative
CD3+
CD8 BV570-A
CD4 BV750-A
Treg
CD4+
CD127 APC-R700-A
CD25 PE-A
1.Naïve (CD8+CCR7+CD45RA+)
2.Effector Memory RA/EMRA (CD8+CCR7-CD45RA+)
3.Central Memory/CM (CD8+CCR7+CD45RA-)
4.Naïve (CD4+CCR7+CD45RA+)
5.Central Memory/CM (CD4+CCR7+CD45RA-)
7.Effector Memory RA/EMRA (CD4+CCR7- CD45RA+)
8. NK CD16+CD56dim
9. NK CD56bright
10. NK CD16+CD56-

(D)

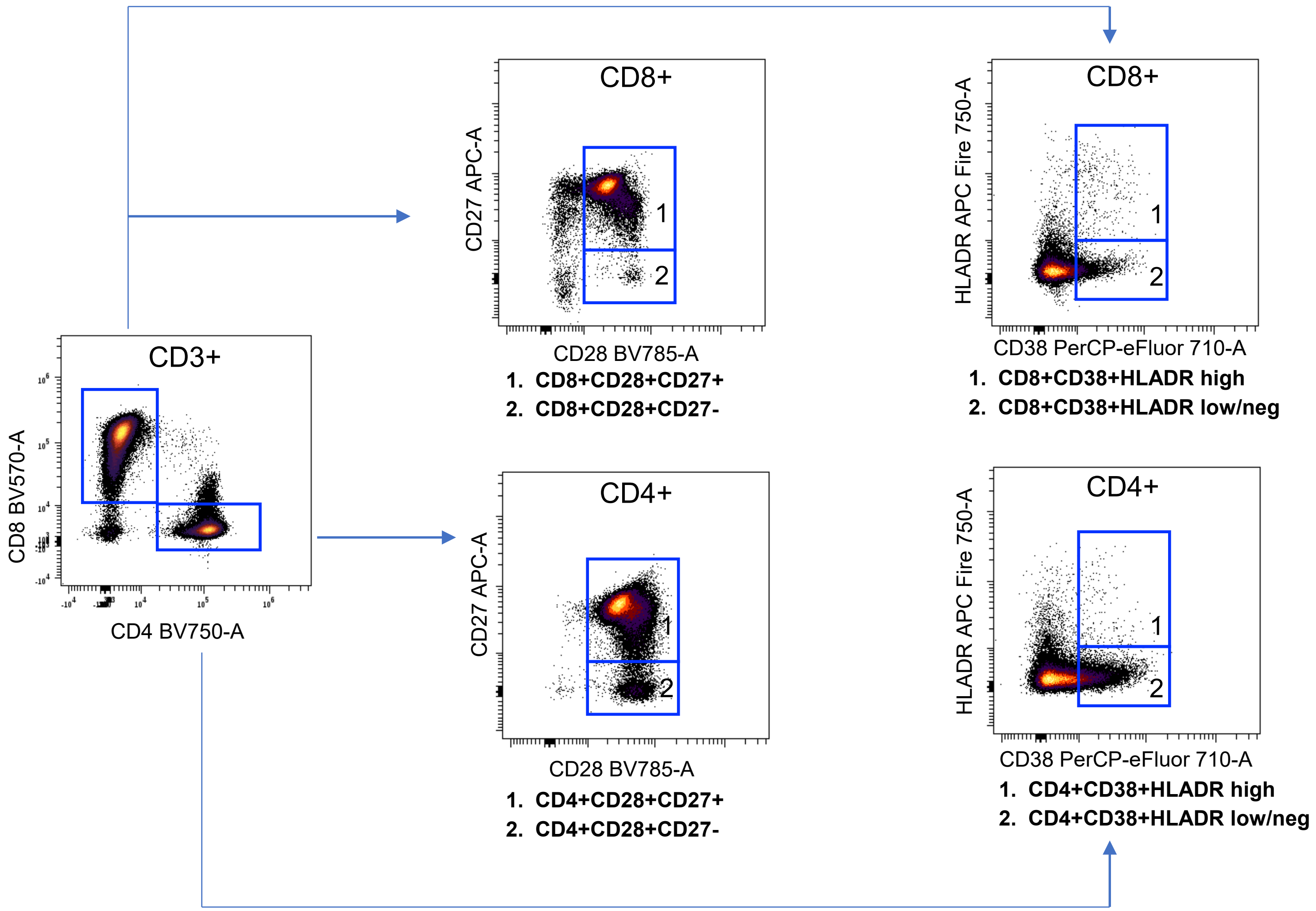
CD3+
CD8 BV570-A
CD4 BV750-A
CD8+
CD27 APC-A
CD28 BV785-A
1
2
1. CD8+CD28+CD27+
2. CD8+CD28+CD27-
CD8+
HLADR APC Fire 750-A
CD38 PerCP-eFluor 710-A
1
2
1. CD8+CD38+HLADR high
2. CD8+CD38+HLADR low/neg
CD4+
CD27 APC-A
CD28 BV785-A
1
2
1. CD4+CD28+CD27+
2. CD4+CD28+CD27-
CD4+
HLADR APC Fire 750-A
CD38 PerCP-eFluor 710-A
1
2
1. CD4+CD38+HLADR high
2. CD4+CD38+HLADR low/neg

Supplemental Figure S3
Validation of CD4+ and CD8+ T cell proportions by multicolor spectral flow cytometry with clinical flow cytometry and RNA sequencing.

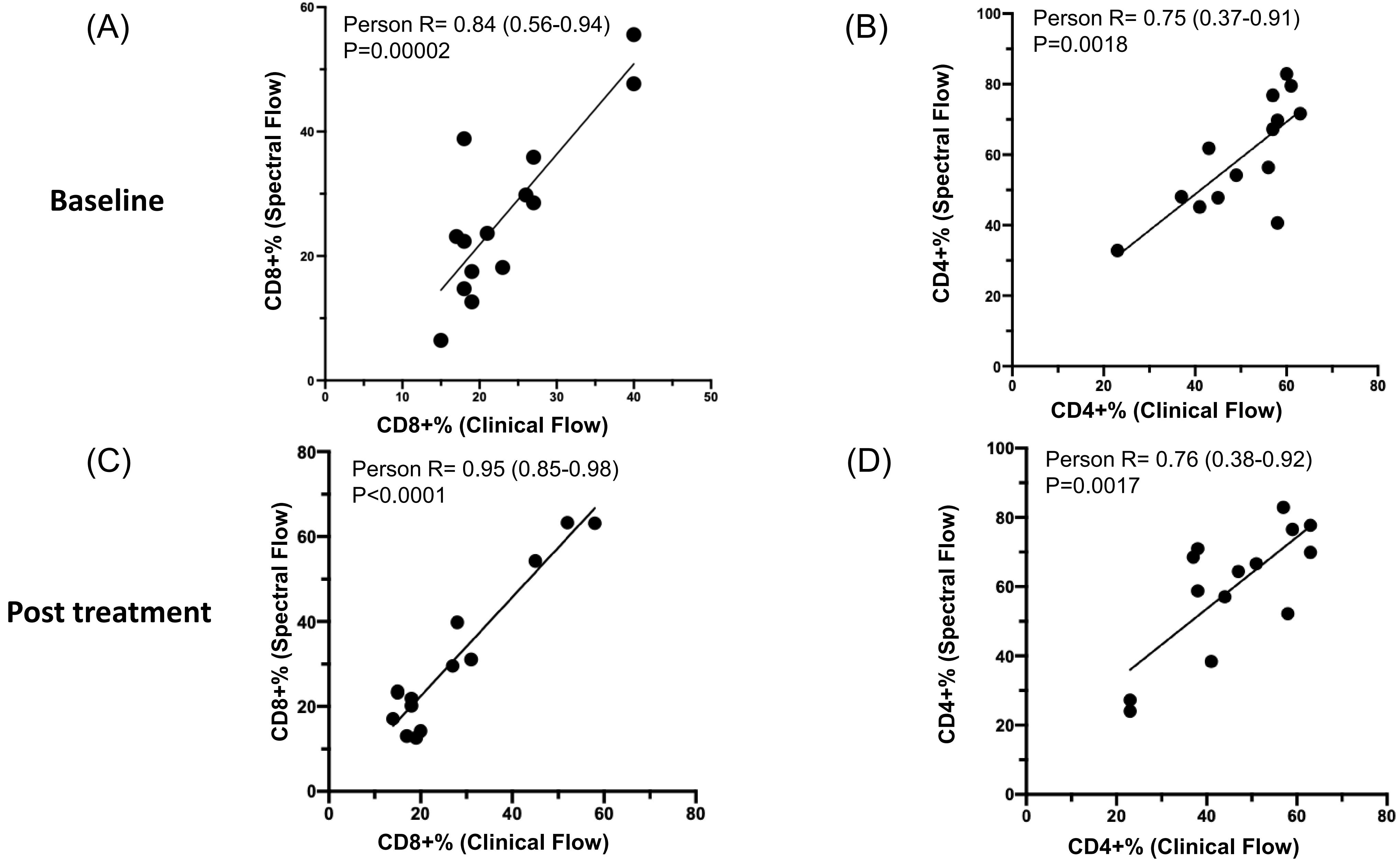

(E)

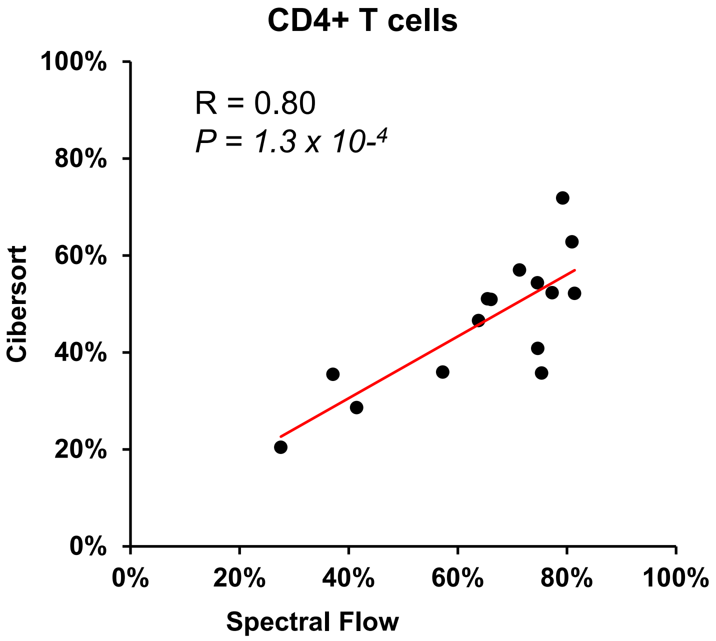
CD4+ T cells
R = 0.80
P = 1.3 x 10-4
Cibersort
100%
80%
60%
40%
20%
0%
0%
20%
40%
60%
80%
100%
Spectral Flow

(F)

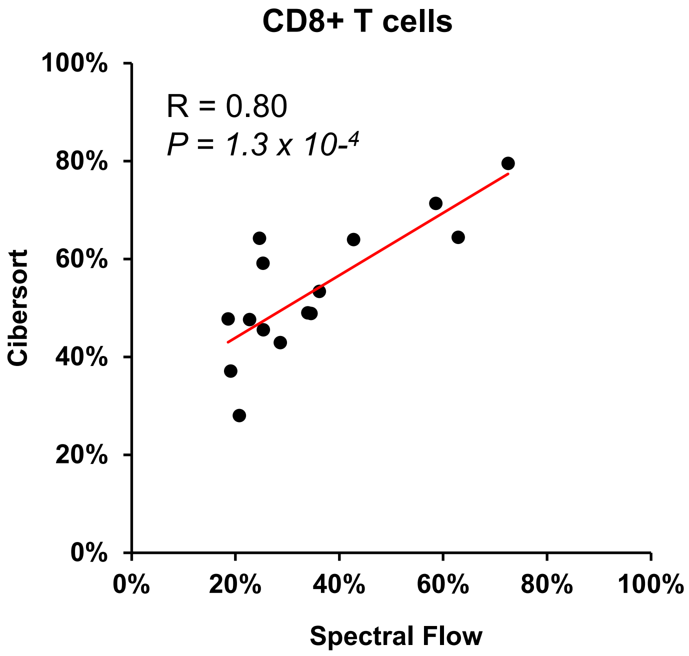
CD8+ T cells
R = 0.80
P = 1.3 x 10-4
Cibersort
100%
80%
60%
40%
20%
0%
0%
20%
40%
60%
80%
100%
Spectral Flow

Supplemental Figure S4. ROC analysis for pertinent immune cell subtypes

(A) CM (CCR7+CD45RA-) CD4+ T cells (baseline)

(B) DN (CD4-CD8-CD3+) T cells (baseline)

(C) CD16+CD56dim NK difference (post-treatment)

(D) CD14+HLADRhigh difference (post-treatment)

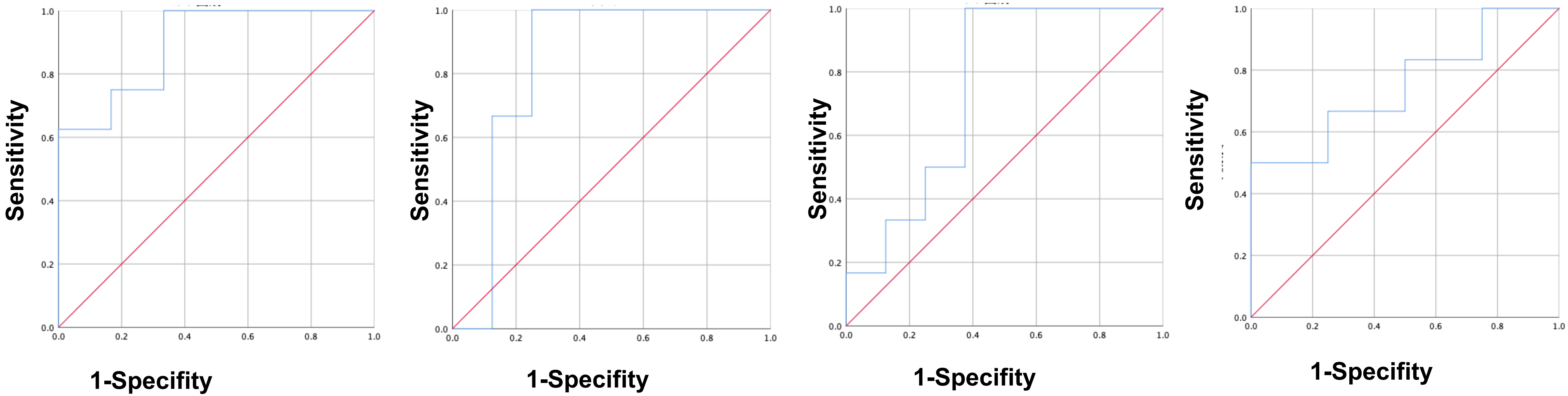

(A)

Area	0.911
Std. Error	0.075
95% confidence interval	0.76-1
P value	0.008

(B)

Area	0.833
Std. Error	0.122
95% confidence interval	0.59-1
P value	0.039

(C)

Area	0.75
Std. Error	0.136
95% confidence interval	0.48-1
P value	0.12

(D)

Area	0.75
Std. Error	0.14
95% confidence interval	0.475-1
P value	0.12

Table S1. Summary of antibodies and dyes used for multi-color spectral flow cytometry

Specificity	Fluorochome	Catalog #	Vendor	Clone
CCR7	BV421	353208	BioLegend	G043H7
CD19	SB436	62-0199-42	Thermo Fisher	HIB19
CD16	eFluor 450	48-0168-42	Thermo Fisher	CB16
TCRγδ	BV480	566076	BD	B1
CD14	BV510	301842	BioLegend	M5E2
CD8	BV570	301038	BioLegend	RPA-T8
CD1c	BV605	331538	BioLegend	L161
PD-1	BV650	329950	BioLegend	EH12.2H7
CD56	BV711	318336	BioLegend	HCD56
CD4	BV750	566355	BD	SK3
CD28	BV785	302950	BioLegend	CD28.2
CD11c	BB515	564490	BD	B-ly6
CD45RA	Alexa 488	304114	BioLegend	HI100
CD3	Alexa 532	58-0038-42	Thermo Fisher	UCHT1
CD25	PE	356104	BioLegend	M-A251
IgD	PE-Dazzle 594	348240	BioLegend	IA6-2
CD95	PE-Cy5	305610	BioLegend	DX2
CD11b	PerCP-Cy5.5	301328	BioLegend	ICRF44
CD38	PerCP-eFluor 710	46-0388-42	Thermo Fisher	HB7
CD57	PE-Cy7	359624	BioLegend	HNK-1
CD27	APC	337169	BD	L128
CD123	Alexa 647	306024	BioLegend	6H6
CD127	APCR-700	565185	BD	HIL-7R-M21
HLA-DR	APC-Fire 750	307658	BioLegend	L243

Table S2. Baseline level of immune cell subgroups

Cell Marker	Patients (N=14)		Normal control (N=4)		P value
	Percent (%)	SD (%)	Percent (%)	SD (%)	
Live	88.3	18.7	99.0	0.2	0.24
Monocytes	26.8	18.6	20.4	2.4	0.57
**CD14+CD16-**	**62.7**	**25.6**	**24.0**	**10.5**	**0.02**
CD14+HLADRhigh	56.5	32.5	80.8	4.1	0.19
CD14+HLADRlow	5.1	5.6	1.0	0.6	0.17
Lymphocytes	45.7	20.2	58.9	2.5	0.23
CD3+	48.1	24.6	71.8	8.3	0.06
CD4+	59.6	15.6	46.9	11.5	0.19
CD4 EM	19.1	15.7	15.7	11.2	0.69
CD4 CM	30.7	11.8	28.9	5.9	0.77
Tregs	11.0	6.3	6.9	1.2	0.22
CD8+	26.8	13.8	36.1	9.6	0.13
**CD8 naive**	**9.5**	**8.3**	**56.2**	**23.2**	**0.001**
**CD8 CM**	**31.7**	**14.4**	**16.3**	**6.1**	**0.03**
CD8 TEMRA	27.3	15.5	14.1	14.9	0.16
CD3-	48.3	26.0	25.6	7.6	0.13
NK CD16+CD56dim	19.8	14.8	35.1	10.7	0.13
NK CD56bright	1.4	1.6	1.3	0.3	0.45
NK CD16+CD56-	3.9	3.3	2.9	0.9	0.65
**LINEAGE NEGATIVE**	**48.0**	**26.8**	**20.6**	**3.2**	**0.03**
Lineage Negative HLADR High	15.6	14.9	11.9	4.1	0.99
**DN**	**6.5**	**7.8**	**13.3**	**1.6**	**0.048**
CD11c HLADR+ Mono	77.9	27.7	89.7	3.1	0.95
NK CD16+CD56+ PD1+ CD27-	25.1	14.9	11.4	4.2	0.13
CD8+ PD1+	16.0	10.8	12.4	4.8	0.96
CD4+ PD1+	15.8	10.3	11.3	6.5	0.28
CD8+CD38+	9.1	7.8	8.2	4.4	0.97
CD4+CD38+	11.8	7.6	20.3	8.2	0.08
CD8+CD28+	50.9	26.9	77.6	21.4	0.06
CD8+CD27+CD28+	42.4	26.6	75.4	22.4	0.08
CD8+CD27-CD28+	8.5	5.8	2.3	1.2	0.06
CD4+CD27+CD28+	75.3	20.9	88.1	10.8	0.23
CD4+CD27-CD28+	18.2	16.4	9.4	6.8	0.38
CD4+CD28+	93.5	7.5	97.6	4.0	0.19
**B cells**	**21.9**	**14.1**	**40.6**	**10.1**	**0.03**

Table S3. Summary of all flow cytometry data with paired samples.

Cell Marker	Before treatment					After treatment				
	Good response (N=11)		Poor response (N=3)		P value	Good response (N=11)		Poor response (N=3)		P value
	Percent (%)	SD (%)	Percent (%)	SD (%)		Percent (%)	SD (%)	Percent (%)	SD (%)	
Live	86.8	20.9	93.9	5.1	0.99	86.0	22.4	72.5	34.8	0.52
Monocytes	25.6	20.6	31.2	8.6	0.88	29.3	19.1	20.8	1.9	0.37
CD14+CD16-	57.1	25.8	83.1	10.9	0.09	64.5	17.9	66.3	41.2	0.91
Lymphocytes	45.2	21.4	47.6	18.7	0.99	41.4	15.4	34.7	17.9	0.66
CD3+	45.7	23.2	57.1	33.1	0.49	46.3	18.9	57.9	20.0	0.37
CD4+	58.3	15.7	64.6	17.0	0.55	57.0	19.2	69.4	12.3	0.32
CD4 EM	20.1	17.1	15.2	10.4	0.65	17.9	15.0	18.2	13.6	0.97
CD4 CM	30.1	12.7	34.9	5.9	0.37	23.7	10.0	30.5	17.0	0.38
Tregs	9.7	4.3	15.9	11.0	0.14	9.6	5.7	15.2	5.2	0.15
CD8+	28.4	15.2	21.0	3.1	0.43	33.7	18.9	18.8	5.6	0.21
CD8 naive	10.1	9.3	7.4	3.9	0.63	8.5	8.2	8.7	9.0	0.96
CD8 CM	28.2	13.1	44.4	13.6	0.09	27.4	16.0	32.9	6.0	0.58
CD8 TEMRA	29.1	15.1	21.1	18.7	0.45	33.4	20.3	26.0	21.1	0.59
CD3-	51.6	23.9	36.3	35.5	0.39	48.3	21.2	38.5	16.7	0.47
NK CD16+CD56dim	18.0	15.2	31.8	4.3	0.37	32.0	13.4	23.7	3.6	0.29
NK CD56+ Bright	1.4	1..69	1.5	1.5	0.66	2.3	3.0	0.8	0.7	0.23
NK CD56- CD16+	4.0	3.7	3.6	0.6	0.88	5.8	4.7	1.2	0.7	0.13
LINEAGE NEGATIVE	49.8	29.9	41.2	11.2	0.64	31.9	17.5	34.6	22.8	0.83
Lineage Negative HLADR high	15.4	16.8	16.0	4.9	0.95	22.1	18.1	29.9	27.1	0.56
DN T cells	5.8	5.8	9.4	14.6	0.5	5.1	4.8	23.0	31.3	0.07
CD11c HLADR+ Mono	74.2	30.2	91.7	5.7	0.35	79.1	25.8	60.3	17.7	0.26
CD8+ PD1+	13.6	9.8	25.0	11.0	0.13	20.9	22.5	9.3	8.0	0.41
CD4+ PD1+	14.3	10.7	21.3	7.8	0.32	16.7	15.6	16.6	17.7	0.99
B cells	20.3	14.4	28.1	13.2	0.42	22.3	15.8	15.6	8.8	0.5
CD14+HLADR-	5.7	6.2	2.8	0.6	0.74	6.8	13.1	7.1	11.3	0.75
CD14+HLADR+	50.5	34.2	78.3	10.3	0.29	56.9	33.6	43.0	37.1	0.37
CD8+CD38+	10.4	8.4	4.4	0.8	0.55	9.2	10.2	8.4	4.9	0.66
CD8+CD38+HLADR-	4.0	3.0	2.1	0.9	0.29	4.5	5.5	3.7	1.4	0.55
CD8+CD38+HLADR+	6.4	8.2	2.3	1.5	0.25	4.7	5.2	4.7	3.5	0.87
CD4+CD38+	10.4	6.3	17.1	11.3	0.23	9.7	7.6	10.3	7.3	0.99
CD4+CD38+HLADR+	2.7	1.9	1.8	0.8	0.55	2.7	2.3	2.3	2.4	0.99
CD4+CD38+HLADR-	7.7	5.9	15.3	12.0	0.36	7.0	6.2	8.0	6.1	0.88
CD8+CD28+	46.4	27.8	67.5	18.0	0.29	45.3	27.5	54.3	26.8	0.66
CD8+CD27+CD28+	37.0	26.4	62.0	19.7	0.23	34.2	24.3	43.9	31.4	0.66
CD8+CD27-CD28+	9.4	6.3	5.4	2.2	0.36	11.1	14.9	10.4	4.8	0.37
CD4+CD27+CD28+	70.5	21.2	93.0	1.7	0.09	74.5	17.7	79.8	12.7	0.99
CD4+CD27-CD28+	21.5	17.1	5.9	0.7	0.06	15.3	13.0	16.6	10.8	0.77
CD4+CD28+	92.0	7.9	98.9	1.1	0.12	89.8	10.5	96.4	2.3	0.77
